# Obstructive sleep apnea treatment improves temporomandibular disorder pain

**DOI:** 10.1007/s11325-023-02883-4

**Published:** 2023-07-25

**Authors:** Anna Alessandri-Bonetti, Frank Lobbezoo, Gilda Mangino, Ghizlane Aarab, Patrizia Gallenzi

**Affiliations:** 1 Department of Dental Clinic and Maxillofacial Surgery, A. Gemelli University Policlinic IRCCS, Catholic University of Sacred Heart, Largo Agostino Gemelli 1, 00168 Rome, Italy; 2 Department of Oral Health Practice, College of Dentistry, University of Kentucky, Lexington, KY USA; 3 Department of Orofacial pain and Dysfunction, Academic Centre for Dentistry Amsterdam (ACTA), University of Amsterdam and Vrije Universiteit Amsterdam, Amsterdam, the Netherlands

**Keywords:** Obstructive sleep apnea, Sleep disordered breathing, Temporomandibular disorders, Headache, Orofacial pain, Facial pain

## Abstract

**Purpose:**

The existence of a bidirectional relationship between poor sleep and pain intensity has been studied, and good sleep quality has been found to be a key factor underlying pain control. The purpose of this prospective cohort study was to observe if OSA treatment provides a reduction in temporo-mandibular disorder (TMD) pain and headache attributed to TMD in patients with obstructive sleep apnea (OSA) after 18 months of OSA treatment.

**Methods:**

A prospective cohort study was conducted on consecutive patients suffering from OSA. Patients underwent polysomnography and TMD examination according to the DC/TMD protocol at baseline and after 18 months. Intensity of TMD pain and headache attributed to TMD were analyzed.

**Results:**

Of 40 patients (31 men, mean age 51.3 ± 10.3 years), 33 underwent OSA treatment. At the follow-up examination after 18 months, significant improvements in the intensity of pain-related TMD and headache attributed to TMD were observed (*p* < 0.05). Seven patients did not start treatment for OSA or discontinued treatment. These patients did not show any significant difference in intensity of TMD-pain or headache attributed to TMD after 18 months (*p* > 0.05).

**Conclusions:**

Significant reductions in intensity of pain-related TMD and headache attributed to TMD were observed in patients with OSA after 18 months of OSA treatment onset, while no difference was observed in subjects not undergoing or discontinuing OSA treatment.

**Trial registration:**

The protocol was registered on ClinicalTrials.gov database with number NCT04948541.

## Introduction

Many efforts have been made in order to understand pain and pain intensity [1]. Good sleep quality has been found to be a key factor underlying pain control [2]. The existence of a bidirectional relationship between pain sensitivity and poor sleep has been shown, suggesting that poor sleep may be responsible for a higher pain sensitivity including in the orofacial area [3]. Some of the most common disorders in the orofacial area are temporo-mandibular disorders (TMDs), which are described as pain and/or dysfunction affecting temporomandibular joints (TMJs), masticatory muscles, or both [4]. TMDs may also be associated with headache secondary to TMD [5].

TMDs have recently been demonstrated to be present with a higher prevalence in patients suffering from obstructive sleep apnea (OSA) compared to healthy controls [6]. OSA is a common sleep-related breathing disorder which affects 9–49% of the general population [7, 8]. It is characterized by episodes of partial or complete upper airway (UA) obstruction despite continuing respiratory efforts [9], which disrupts normal sleep architecture and leads to micro-arousals throughout the night [10]. Behavioral therapy (e.g., weight loss, controlling sleep position) [11–13], continuous positive airway pressure (CPAP) [14], mandibular advancement devices (MAD) [15–17], and UA surgery [18, 19] are therapeutic options that exert their effect by preventing UA closure during sleep. When untreated, OSA is associated with increased morbidity and mortality [20, 21].

A higher prevalence of TMDs has been shown in patients with OSA [6]. However, no studies have examined if a reduction of pain-related TMDs may be observed when OSA is successfully managed. Since sleep fragmentation may lead to increased pain perception [22], an improvement in pain could be expected after OSA treatment. The literature provides insufficient evidence regarding the relationship between TMDs and OSA [23]. Therefore, the aim of this study was to observe if OSA treatment provides a reduction in the intensity of TMD pain and headache attributed to TMD in OSA patients after 18 months of OSA treatment. The hypothesis was that patients undergoing OSA treatment would show a reduction in pain compared to pre-treatment values, while no significant differences would be observed in subjects not undergoing or discontinuing treatment.

## Material and methods

This prospective study was conducted at the A. Gemelli Hospital (Italy). Approval of the study was obtained by the Ethics Committee of the A. Gemelli Foundation with the protocol number 3928/21, prior to the start of the trial. The protocol was registered on ClinicalTrials.gov database with number NCT04948541.

Consecutive adult patients, who had been diagnosed with OSA and were referred for OSA management to the department of otorhinolaryngology, were invited to participate in the study between May 2018 and July 2019. All patients, independently of the type treatment offered, underwent a complete TMD examination prior to start of the treatment for OSA and were invited to undergo a second examination 18 months after the first evaluation.

Subjects were included if they were ≥ 18 years old and presented with an apnea–hypopnea index (AHI) of ≥ 5 determined by a polysomnographic study (PSG) as recommended by the American Academy of Sleep Medicine guidelines [24], prior to start of the treatment. Exclusion criteria were: patients suffering from painful dental problems of non-musculoskeletal origin that would alter their pain perception, patients using medications that would alter their pain perception, patients presenting with cognitive impairment, and patients who did not give informed consent.

In order to measure intensity of pain-related TMD and headache attributed to TMD, the Italian version of the Diagnostic Criteria for Temporo-Mandibular Disorders (DC/TMD) [25] were used for both TMD examinations. The DC/TMD protocol represents the gold standard for TMD diagnosis for both clinical and research purposes and consists of several questionnaires which are administered to patients, and a clinical examination conducted by a calibrated examiner. The same researcher was responsible for all examinations. The TMD diagnoses were obtained according to the DC/TMD diagnostic decision tree. Data regarding TMD incidence and prevalence of pain-related TMD and headache attributed to TMD both in patients undergoing therapy and in patients not undergoing/not pursuing therapy were recorded. In addition, all patients underwent a complete medical and dental examination, during which body mass index (BMI) was also recorded.

The graded chronic pain scale was administered to patients as described in the DC/TMD protocol. A numeric pain scale was used, where 0 would indicate “no pain” and 10 would indicate “pain as bad as it could be”, and patients were asked to provide values indicating “pain right now”, “worst pain in the last 30 days”, and “average pain in the last 30 days”. Values obtained were computed and multiplied by 10, thus obtaining a value indicating Characteristic Pain Intensity (CPI) with values going from 0 to 300.

A numeric pain scale was also used to assess interference of pain. Zero would indicate “no interference” and 10 would indicate “unable to carry on any activities”. Interference of pain with “usual activities in the last 30 days”, interference of pain in “recreational, social and family activities in the last 30 days”, and interference of pain in “ability to work in the last 30 days” were recorded, and values obtained were computed and multiplied by 10 obtaining a value indicating points for pain-related interference score with values going from 0 to 300. The number of days in which the patient was unable to perform “usual activities in the last 30 days because of facial pain” was also recorded, thus obtaining a value indicating points for disability days. Total disability points were obtained by adding up the points for disability days and those for interference score. CPI grade was calculated according to the DC/TMD scoring protocol [25]; therefore, considering both CPI values and disability points with grade 0 in the presence of CPI of 0; grade 1 with CPI ≤ 50 and disability points < 3; grade 2 with CPI ≥ 50 and disability points < 3; grade 3 with any CPI and disability points 3–4; grade 4 with any CPI and disability points 5–6. [26, 27]. CPI was used as a measure for TMD-pain intensity and any change in CPI grade between baseline and follow-up was considered to be significant.

### Statistical methods

A power calculation was performed using a mean value of change in pain from pre- to post-OSA treatment (1.75 points change on a pain numeric rating scale, SD 1.06) [28]. Based on those values, a sample size of 6 would provide a power of 80% to detect a pre-post intervention change using a paired *t*-test with a significance level of 0.05. Demographic data are presented as mean and standard deviation, and categorial variables are presented as percentage. Data distribution was tested with Shapiro–Wilk test. Since data were normally distributed, paired *t*-test was used to assess the difference between the first and second examination in both treated and untreated groups. Comparison between treated and untreated patients was performed with independent *t*-test. IBM SPSS Statistics for Mac, Version 27.0 was used for statistical analysis; all were conducted with 95% confidence interval. Probability levels of *P* < 0.05 were considered statistically significant.

## Results

**Fig. 1:**
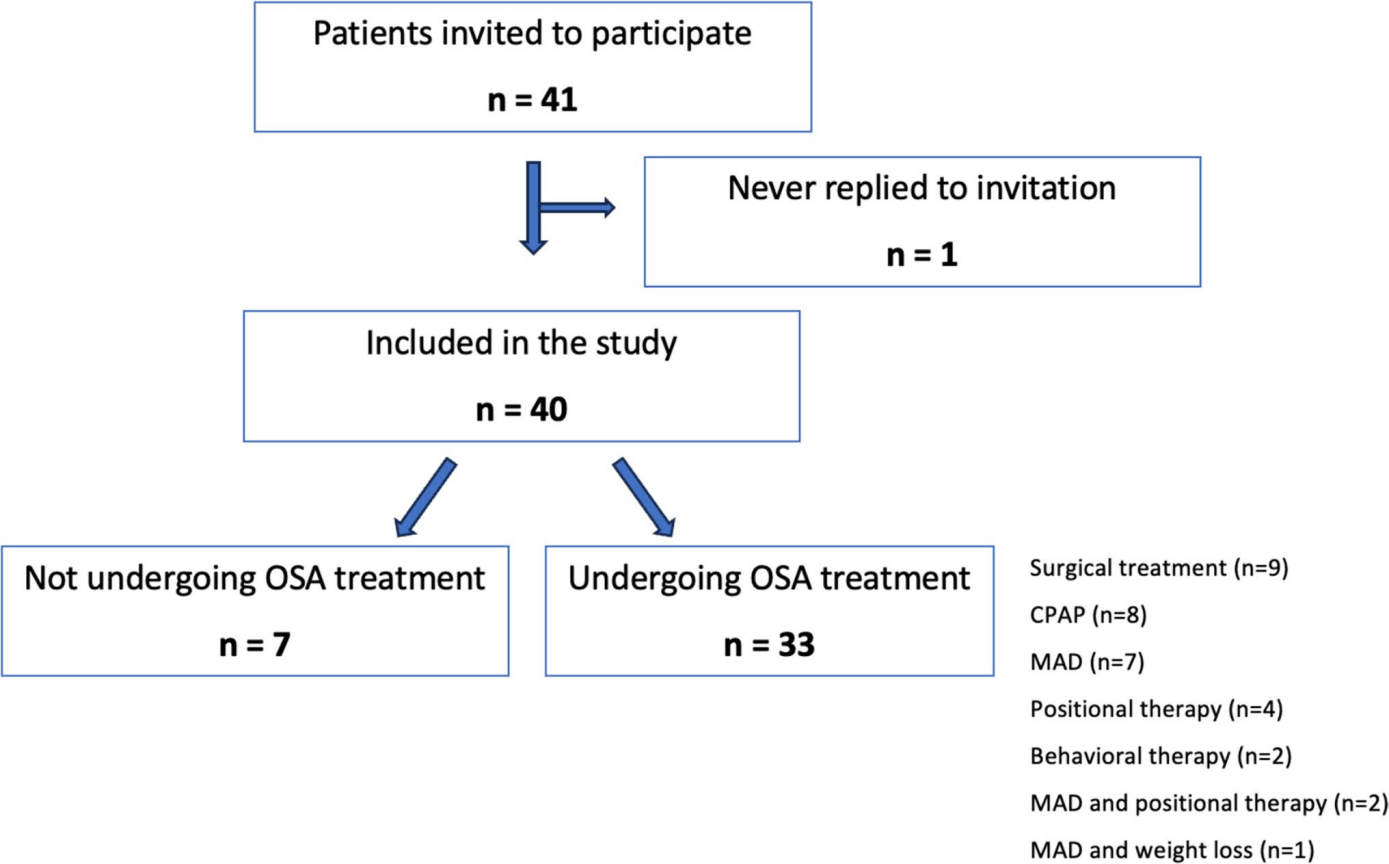
Patients distribution through the study

Of the 41 patients invited to participate in the study, 40 accepted the invitation and underwent the two TMD examinations. A total of 40 patients were included in the analysis consisting of 31 men with a mean age of 51.3 ±  10.3 years. The characteristics of all included patients are shown in Table 1. Patients’ distribution through the study is shown in Fig. 1.

**Table 1:** Characteristics of subjects undergoing OSA treatment and not undergoing/not pursuing treatment, both at first and second examination. Values are presented as percentage or as means ± standard deviation

	Undergoing treatment	Undergoing treatment	Not undergoing/not pursuing treatment	Not undergoing/not pursuing treatment
	Pre-treatment	Post-treatment	Baseline	Follow-up
Number of patients, n (%)	33 (82.5%)	33 (82.5%)	7 (17.5%)	7 (17.5%)
Age (years)	49.8 ± 10.7	51.4 ± 10.4	48.6 ± 10.5	50.6 ± 10.1
Male/female, n	27/6	27/6	4/3	4/3
Apnea hypopnea index, (Ev/h)	21.9 ± 13.1*	8.4 ± 4.7*	13.2 ± 7.4	/
Body mass index (kg/m^2^)	26.5 ± 3*	25.4 ± 3.2*	25 ± 4.8	24.8 ± 3.8
pTMD and/or headache attributed to TMD, N (%)	11 (33.3%)*	8 (24.2%)*	3 (42.9%)	4 (54.1%)

*n*, number; *Ev/h*, number of events per hour; *OSA*, obstructive sleep apnea; *pTMD*, pain-related temporo-mandibular disorders;

^*^ = significant difference: *p* < 0.05, comparison between pre- and post-treatment

### Patients undergoing 18 months of OSA treatment

Of the 40 patients enrolled, 33 (82.5%), 27 men, underwent one or more treatments for OSA. Of these, 9 patients underwent surgical procedures (5 underwent uvulopalatoplasty and 4 underwent nasal surgery), 8 used CPAP, 7 used MAD, 4 used positional therapy, 2 underwent behavioral therapy including significant weight loss, 2 used both MAD and positional therapy, and 1 underwent significant weight loss and used MAD. Independent of the therapeutic option, OSA therapy was defined as successful by a certified sleep physician, in case of a reduction in AHI of at least 50% to fewer than 20 Ev/h [29]. All patients had undergone a second sleep study demonstrating success of the therapy, with a mean AHI reduction from 21.9 ± 13.1 to 8.4 ± 4.7 (*p* < 0.05). In the treated sample, BMI went from 26.5 ± 3 to 25.4 ± 3.2 (*p* < 0.05). The presence of pain-related TMD and headache attributed to TMD diagnosis in patients with OSA undergoing treatment for at least 18 months was 24.2%.

**Fig. 2:**
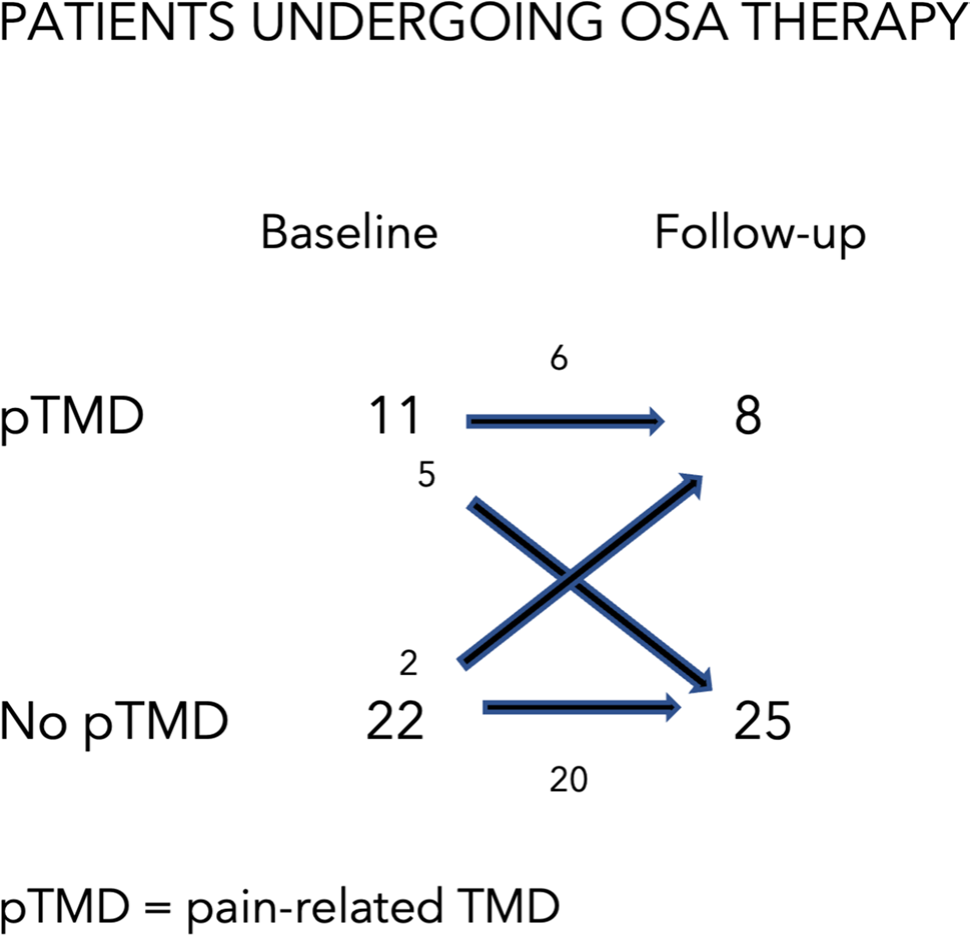
Incidence and prevalence of TMD pain (pTMD) and headaches attributed to TMD in patients undergoing OSA treatment

Of the 33 patients who underwent treatment for OSA, 11 presented pain-related TMD prior to the start of treatment. After 18 months of treatment, 8 of these 11 presented a significant improvement in pain intensity. A reduction in pain intensity was observed in 3 patients (one patient went from CPI grade 4 to CPI grade 2, one from grade 4 to grade 3, and one from grade 2 to grade 1), while 5 patients no longer experienced pain (4 of them went from grade 1 to grade 0, and one patient went from grade 2 to grade 0). Three of the 11 patients with pain-related TMD remained in the same CPI grade compared to before starting the treatment (one patient remained in grade 1, and 2 patients remained in grade 2). The difference in CPI grade between pre-treatment and during treatment was found to be statistically significant (*p* < 0.05). After 18 months of OSA treatment, of the 22 patients with no pain-related TMDs at the initial visit, 20 patients remained negative at the follow-up examination, while 2 patients developed pain-related TMD during treatment, both going from CPI grade 0 to grade 1 (Fig. 2).

Significant differences were found in pain-related TMDs’ intensity between the treated and untreated patients, as a reduction in CPI grade was observed only in treated patients.

### Patients not undergoing/not pursuing OSA treatment

**Fig. 3:**
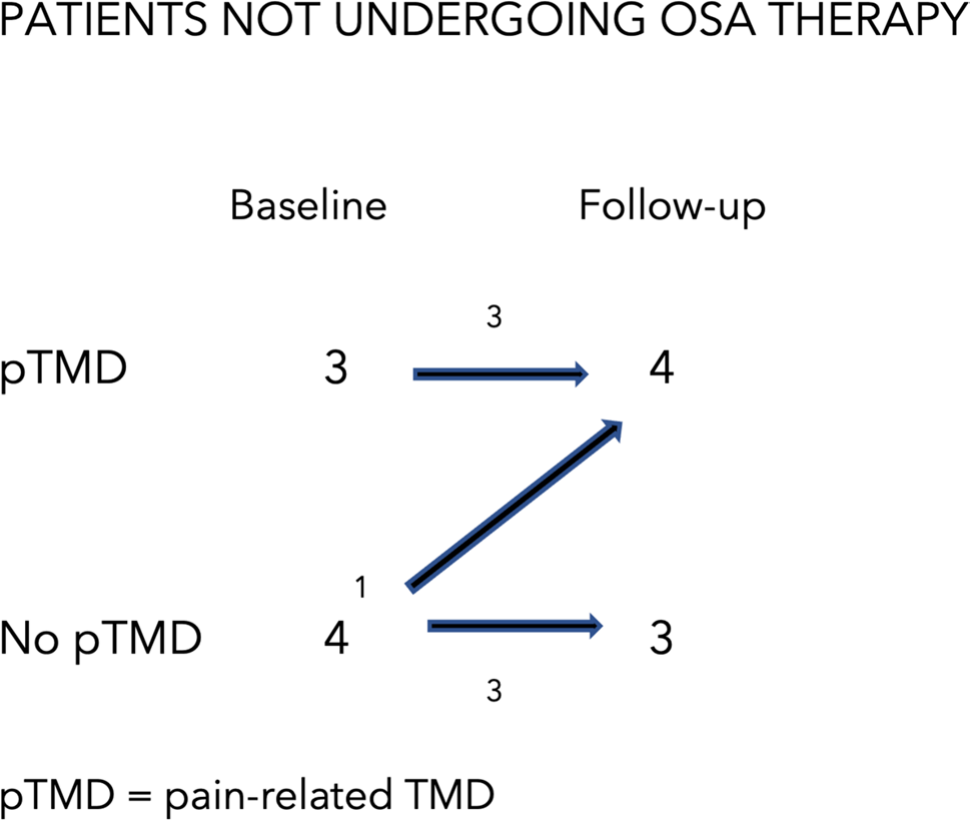
Incidence and prevalence of pTMD and headaches attributed to TMD in patients not undergoing OSA treatment

Seven patients (17.5%), 4 men, did not start treatment for OSA, or they discontinued it. The main reason for discontinuing the treatment was the discomfort of the therapy/device. When observing this population, 3 out of 7 patients presented pain-related TMD diagnoses according to the DC/TMD protocol at the initial evaluation. Of these, one patient did not present a difference in CPI grade at the follow-up visit, one patient reported an improvement of the pain-related TMDs with a reduction in CPI grade, and one patient reported worsening of the pain-related TMDs as evidenced by a higher CPI grade. One patient did not present any pain-related TMD at the time of the initial evaluation, but developed pain before the follow-up visit 18 months later. Of the 7 patients, 3 did not present any pain-related TMD at the time of the initial evaluation and were still negative for pain at the follow-up evaluation (Fig. 3). No statistically significant differences were observed between the two examinations in the untreated sample regarding the intensity of pain-related TMDs as measured by CPI grade (*p* > 0.05). Compared to patients undergoing treatment for OSA, no significant differences were noted regarding their mean age or mean BMI (*p* > 0.05).

## Discussion

In the present study, changes in pain-related TMD and headache attributed to TMD after 18 months of OSA treatment were assessed. The hypothesis was that patients undergoing OSA treatment would yield a reduction in intensity of TMD-pain and headache attributed to TMD compared to pre-treatment values and that no changes would be observed in subjects not undergoing/not pursuing treatment.

This is the first study to evaluate the effect of OSA treatment on pain-related TMDs. The results of the present study show that OSA treatment provides a significant reduction in pain-related TMD and headache attributed to TMD in patients suffering from both OSA and TMD, without providing any treatment for TMD. The present findings are in agreement with a recent study demonstrating that treatment with MAD is beneficial in reducing headache intensity in patients with OSA [28]. The decision of merging all therapeutic options in one sample was based on the need to observe findings depending on OSA treatment in general rather than on a specific therapeutic option, as well as on the knowledge that different therapeutic options may affect TMDs only in the initial period of use, and that these differences are limited with long-term use [30, 31]. The present findings suggest that TMDs should be routinely assessed in the OSA diagnostic and treatment processes, and that OSA treatment may be provided for reducing both OSA and pain-related TMDs in patients presenting both conditions. One could hypothesize that our findings may be due to TMDs fluctuation over time [32] rather than being a result of OSA therapy and, in fact, TMDs fluctuation can explain the incidence of new pain-related TMD cases in both untreated and treated patients. However, the fact that a significant difference was observed in reduction in pain intensity in patients undergoing OSA treatment, while no difference was observed in those not undergoing/not pursuing treatment, suggests that OSA treatments are likely to play a significant role in improving pain-related TMDs in subjects suffering from both these conditions; however, further RCTs are needed to confirm these findings.

The finding that a reduction in pain has been observed in subjects undergoing OSA treatment should not be surprising. In fact, it is known that poor sleep is associated to hyperalgesic changes [33, 34] and that patients suffering from OSA present a higher prevalence of TMDs compared to controls [6]. It has recently been demonstrated that CPAP therapy exerts a significant effect in increasing pain threshold and tolerance [35], supporting once again the association between OSA and pain [36, 37]. Based on the available literature, it is reasonable to hypothesize that the significant improvement observed in our sample was secondary to the improvement in sleep quality obtained during OSA treatment.

The proportion of men to women in the studied sample reflects that of patients with OSA, but not that of patients with TMD. In fact, OSA tends to be more prevalent in men, while TMD is more commonly observed in women [38]. It is known that gender affects pain modulation [39], but due to the limited sample size, no statistical analysis could be conducted to examine if gender represents a significant factor influencing pain improvement. Future RCT studies that control for gender as well as other confounding factors such as smoking, caffeine intake, shifts in work, anxiety and comorbidities are needed to better understand the mechanisms behind the present results.

A reduction in BMI was observed in treated patients. This reduction could have played a double role: on the one hand, it may have allowed for an improvement in OSA and improve clinical outcomes [40, 41]. On the other one, reduction in BMI may have helped to reduce pain perception since body fat has been demonstrated to be associated to musculoskeletal pain in healthy individuals [42].

### Limitations

A limitation of the present study is the small sample size, especially regarding patients not undergoing/not pursuing treatment. Future RCT studies with larger samples should be performed to confirm the present results and better address this subgroup.

Another limitation is the lack of information regarding TMD and sleep treatments during the 18 months until follow-up examination occurred. Patients did not report any treatment at their second examination. However, recall bias cannot be excluded. Therefore future studies are encouraged to provide more frequent follow-ups when examining patients.

Also, the present study lacks information regarding the treatment phase in patients discontinuing treatment. In fact, on the one hand, the treatment phase may be responsible for improvement in pain intensity, while on the other hand, treatment could also cause the development of pain. However, this limitation does not undermine the present results as it has been shown that short-lasting sleep deprivation is associated with increased pain sensitivity [43] and that pain-related TMDs are often self-limiting when the identified source of pain has been removed [44]. This suggests that the treatment phase may have played only a marginal role in results presented.

It has been suggested that patients suffering from OSA, prior to undergoing treatment, should be screened for TMDs [31]; and the present study confirms the importance of a close collaboration between orofacial pain specialists, dental sleep doctors, and sleep physicians. In light of the present study, we suggest that more questions regarding sleep characteristics and quality should be routinely asked to patients undergoing TMD evaluation and, in the presence of sleep disturbances, we suggest addressing these conditions as part of the management of the TMDs.

## Conclusion

Within the limitations of this study, the findings suggest that a significant improvement in pain-related TMD and headache attributed to TMD may be observed in patients with OSA after 18 months of treatment for OSA. This study highlights the strong association between sleep and pain-related TMD, and underlines the need of a close collaboration between orofacial pain specialists, dental sleep doctors, and sleep physicians.

## Data Availability

This manuscript has no associated data.
